# Case Report: Dynamic J-point elevation as a novel precursor to torsade de pointes: electrocardiographic markers for proactive management

**DOI:** 10.3389/fcvm.2025.1613757

**Published:** 2025-11-11

**Authors:** Huihui Zhang, Zhi Zhang, Miaolin Zhang, Hong Yuan

**Affiliations:** 1 Department of Cardiovascular Medicine, First People’s Hospital of Linping District, Hangzhou, Zhejiang, China; 2 Department of Orthopedics, First People's Hospital of Linping District, Hangzhou, Zhejiang, China

**Keywords:** ventricular tachycardia, torsade de pointes, sudden death, early repolarization, electrocardiogram

## Abstract

This case report presents a 41-year-old male who experienced out-of-hospital cardiac arrest (OHCA) and was successfully resuscitated through prehospital emergency medical services and in-hospital advanced cardiovascular life support. Continuous electrocardiographic (ECG) monitoring revealed a unique pattern of J-point and ST-segment elevation in lead II, which preceded the development of torsade de pointes (TdP). The complete ECG evolution, from initial J-point elevation to the onset of polymorphic ventricular tachycardia, was meticulously documented. This rare case provides valuable insights into the electrocardiographic precursors of malignant ventricular arrhythmias and highlights the importance of continuous ECG monitoring in identifying high-risk patients.

## 1 Introduction

Sudden cardiac death (SCD) accounts for approximately 15%–20% of all natural deaths worldwide, with ventricular arrhythmias being the predominant underlying mechanism (1). Despite advances in diagnostic techniques, up to 40% of SCD cases remain unexplained after comprehensive evaluation, highlighting a critical knowledge gap in arrhythmogenic substrates (2). Early repolarization syndrome (ERS) and channelopathies have emerged as important causes of idiopathic ventricular fibrillation, yet their dynamic electrocardiographic manifestations are rarely captured during acute arrhythmic events (3).

The J-wave, the electrocardiographic hallmark of early repolarization, is not a mere electrical curiosity but a manifestation of a profound transmural voltage gradient during the early phase of myocardial repolarization. This gradient arises from a physiological imbalance between the epicardial and endocardial action potentials. At the cellular level, it is primarily driven by an augmentation of the transient outward potassium current or the ATP-sensitive potassium current, which accelerates epicardial repolarization, or a reduction in the depolarizing sodium or L-type calcium currents, which delays endocardial repolarization (4, 5). This imbalance creates a voltage heterogeneity that manifests on the surface ECG as the J-wave or point elevation. The electrophysiological phenotype can stem from diverse etiologies, encompassing both inherited channelopathies (e.g., loss-of-function variants in genes such as *KCNJ8, CACNA1C, CACNB2, and SCN5A*) (6, 7) and acquired conditions such as acute ischemia (which activates potassium current), hypothermia, and electrolyte disturbances (6).

The clinical significance of the J-wave, once considered a benign variant, is now recognized to be nuanced and highly dependent on specific features. Contemporary evidence indicates that dynamic changes (fluctuation in amplitude or morphology, often accentuated by bradycardia), high amplitude (>0.2 mv), and an inferolateral distribution (particularly in the inferior leads) are key features signaling a higher arrhythmic risk (8, 9). The proposed mechanism for arrhythmogenesis is phase 2 reentry, where the accentuated transmural dispersion of repolarization provides the substrate for a reentrant circuit, potentially triggering lethal ventricular fibrillation (10, 11).

This case report presents a 41-year-old male with out-of-hospital cardiac arrest (OHCA) whose in-hospital telemetry documented the complete electrophysiological evolution from J-point elevation to torsade de pointes (TdP), exclusively in lead II. Unlike typical Brugada syndrome or classical ERS patterns, this case demonstrates:

1. A novel spatial distribution of repolarization abnormalities limited to inferior lead (II).
2. A clear temporal progression from saddle-shaped to downsloping ST elevation preceding TdP
3. The profound diagnostic challenges posed by intermittent high-risk ECG patterns

The primary aim of this manuscript is to present this unique electrophysiological sequence to enhance awareness among clinicians. Furthermore, we aim to elucidate the diagnostic approach to unexplained cardiac arrest by detailing the comprehensive workup undertaken. Finally, we discuss the implications of dynamic J-point elevation within the evolving paradigm of J-wave syndromes and explore the challenges of interpreting genetic variants of uncertain significance in this context. By doing so, we hope to contribute to the proactive identification and management of patients with similar elusive, yet potentially lethal, repolarization disorders.

## 2 Case description

### 2.1 Clinical course

The patient was transported by emergency medical services (EMS) after collapsing at work. Prehospital monitoring showed pulseless electrical activity, and advanced cardiac life support (ACLS) was initiated within 5 min of arrest. Continuous ECG during transport revealed no ST-segment deviations or arrhythmias prior to hospitalization.

According to collateral history obtained from family members, the patient experienced a syncopal episode approximately one month prior to the cardiac arrest, which spontaneously resolved without intervention. Although a neurological workup including 24-hour Holter monitoring was performed at that time, no definitive cause was established. In the week leading up to the event, he reported no palpitations, presyncope, or additional syncopal events. His primary cardiovascular risk factor was a significant smoking history, having consumed approximately 10 cigarettes daily for 15 years. He had no known history of hypertension, diabetes, dyslipidemia, or family history of premature cardiovascular disease or hereditary cardiac disorders.

Upon arrival at the emergency department, the patient was unconscious. Vital signs were as follows: blood pressure, 108/65 mmHg under vasopressor support; heart rate, 102 beats per minute; respiratory rate, 16 breaths per minute (mechanically ventilated); oxygen saturation, 98% on mechanical ventilation; and body temperature, 36.5°C. Physical examination revealed no signs of trauma. Cardiopulmonary auscultation revealed normal heart sounds without murmurs and clear lung fields. Neurological examination revealed no focal deficits, and the abdomen was soft without organomegaly. No skin abnormalities or edema were noted.

### 2.2 Electrocardiographic findings

**Figure 1:**
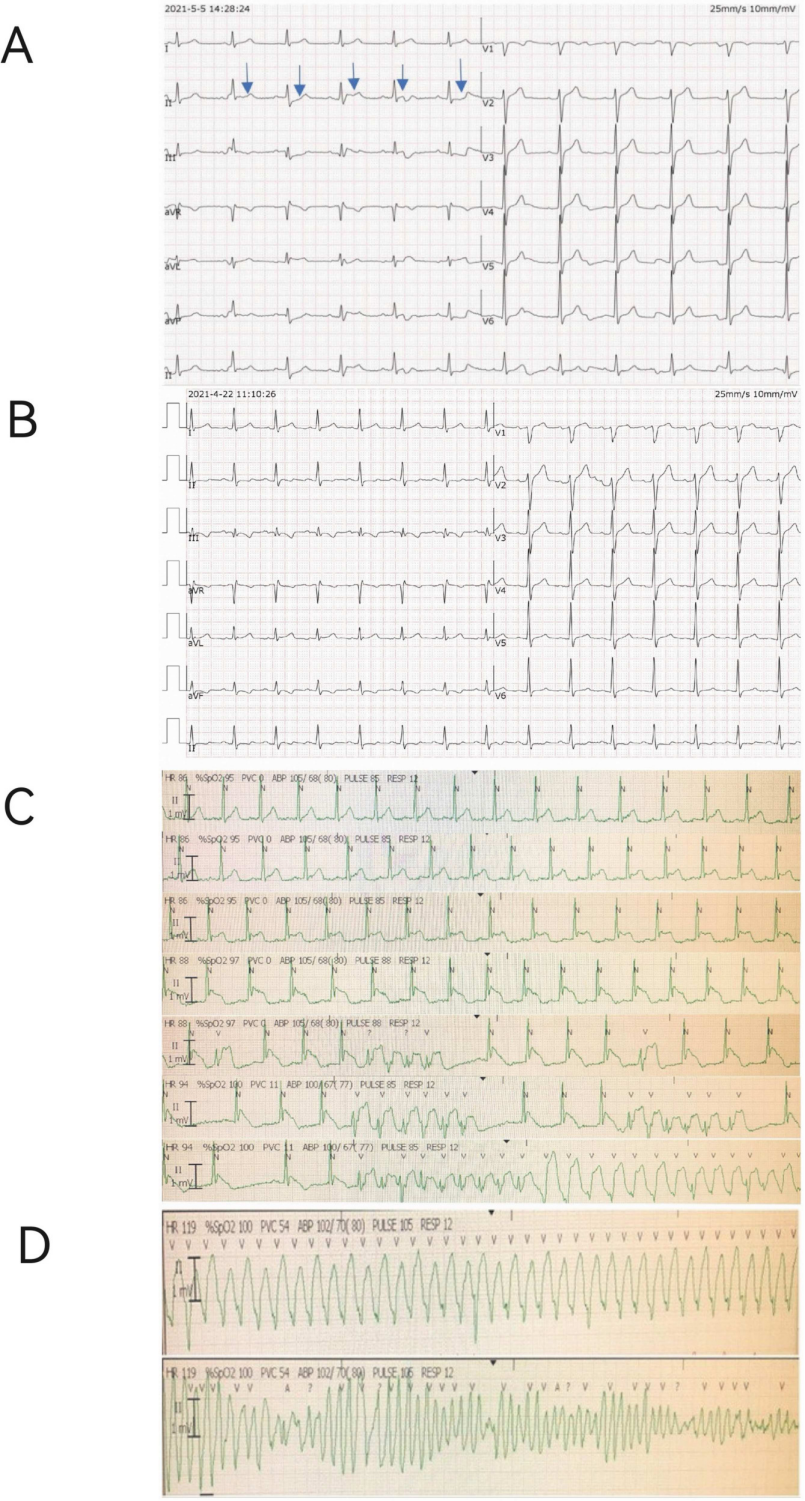
Electrocardiographic (ECG) evolution and monitoring strips. **(A)** Continuous ECG monitoring strip from lead II upon ICU admission, showing significant J-point and ST-segment elevation (blue arrows). **(B)** Baseline 12-lead ECG recorded prior to symptom onset, demonstrating a normal pattern for comparison. **(C)** Sequential ECG strips from ICU monitor showing the progression from gradual J-point and ST-segment elevation (top) to the emergence of ventricular tachycardia (bottom). **(D)** The subsequent development of polymorphic ventricular tachycardia (Torsade de Pointes).

Continuous ECG monitoring revealed significant dynamic changes in the J point and ST-segment of lead II (Figure 1A). Notably, the baseline ECG obtained prior to symptom onset did not demonstrate these pronounced alterations (Figure 1B). During intensive care unit (ICU) monitoring for advanced life support, the patient developed sudden ventricular tachycardia approximately 10 h post-admission. The complete electrophysiological evolution was captured, documenting the progression from gradual J-point and ST-segment elevation in lead II to the development of torsade de pointes (Figures 1C,D). The patient successfully underwent emergency defibrillation for this malignant arrhythmia.

Although continuous telemetry in lead II captured the dynamic electrophysiological progression, simultaneous 12-lead Holter monitoring was not performed, limiting multi-lead analysis during the pre-arrhythmic period.

### 2.3 Diagnostic workup

Initial laboratory investigations revealed elevated high-sensitivity cardiac troponin I (cTnI) at 0.08 ng/mL (reference < 0.04 ng/mL), creatine kinase (CK) at 90.4 U/L (reference 50–310 U/L), creatine kinase-MB (CK-MB) at 48 U/L (reference < 25 U/L), and serum potassium (K⁺) at 3.6 mmol/L (reference 3.5–5.1 mmol/L). Other relevant laboratory parameters were within normal limits, including white blood cell count (8.5 × 10⁹/L), hemoglobin (14.6 g/dL), platelets (227 × 10⁹/L), C-reactive protein (10.8 mg/L), serum sodium (146 mmol/L), creatinine (89 μmol/L), thyroid-stimulating hormone (1.9m IU/L), and magnesium (0.87 mmol/L).

**Figure 2:**
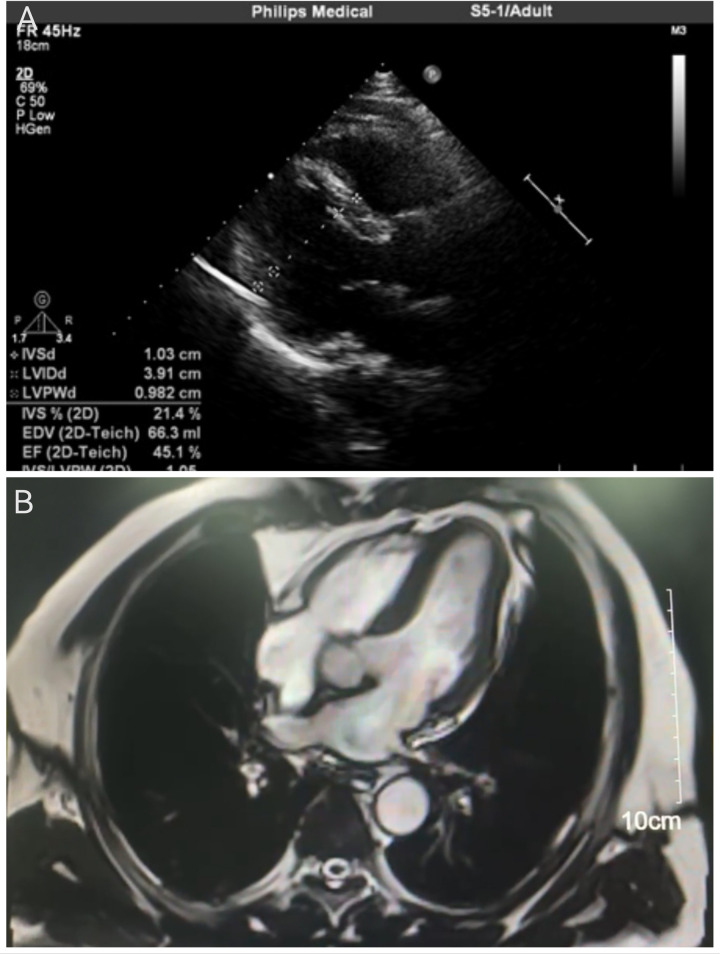
Cardiac imaging assessments. **(A)** Transthoracic echocardiogram (parasternal long-axis view) indicating a structurally normal heart. Quantitative measurements are displayed, including a left ventricular ejection fraction (LVEF) of 45.1%. **(B)** Cardiac magnetic resonance image (four-chamber view) with late gadolinium enhancement. The homogeneous myocardial signal and the scale bar (10 cm) confirm the absence of edema, fibrosis, or structural abnormalities.

**Figure 3:**
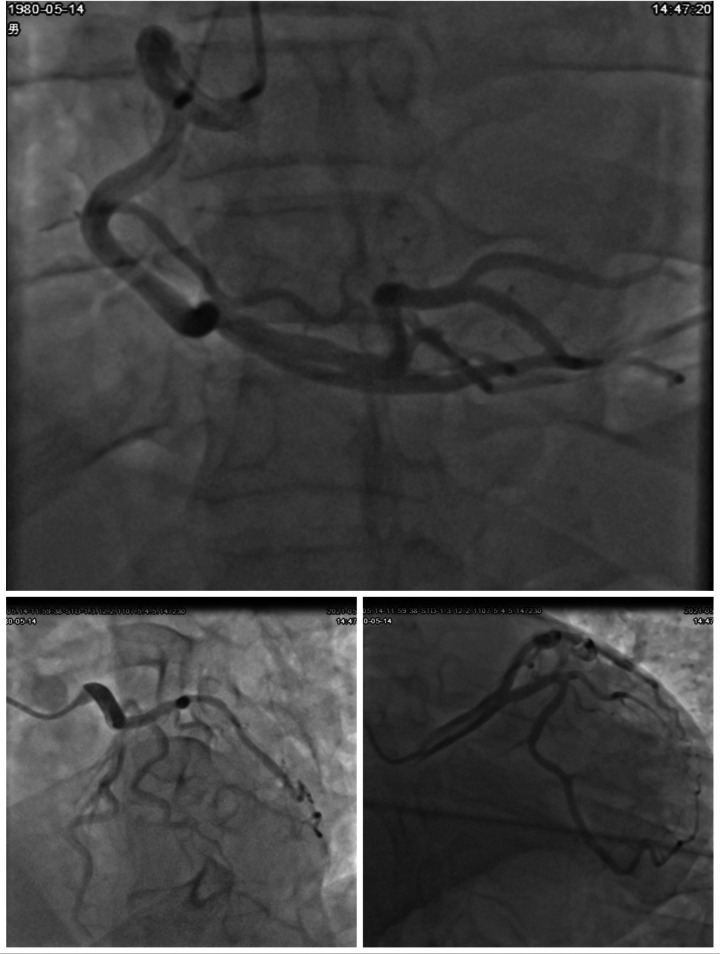
Coronary angiographic findings. Multiple angiographic views of the coronary arteries. The top image shows the right coronary artery (RCA) with its branches. The bottom left image highlights the left main coronary artery and its bifurcations into the left anterior descending (LAD) and left circumflex (LCX) arteries. The bottom right image provides another detailed view of the coronary vasculature. All views demonstrate patent arteries without evidence of stenosis or occlusion.

A comprehensive diagnostic evaluation was performed. Transthoracic echocardiography (Figure 2A) showed a structurally normal heart with a left ventricular ejection fraction (LVEF) of 45.1% and globally reduced systolic function in the absence of regional wall motion abnormalities. Cardiac MRI with late gadolinium enhancement (Figure 2B) confirmed the absence of myocardial edema, fibrosis, or any other structural pathology. Coronary angiography (Figure 3) revealed no evidence of stenosis. An acetylcholine challenge test was negative for coronary vasospasm. Toxicological screening was negative for common QT interval-prolonging medications and for a comprehensive panel of illicit substances (cocaine, amphetamines, cannabinoids, opioids).

### 2.4 Family history and genetic evaluation

A comprehensive three-generation family history revealed no sudden cardiac death, syncope, or premature cardiovascular disease. The patient's 53-year-old brother underwent full cardiovascular assessment (ECG, echocardiography, cardiac MRI), all within normal limits.

Genetic testing employing a 25,701-gene panel was performed for the patient and his brother, with interpretation per ACMG guidelines (12). In the patient, no pathogenic(P) or likely pathogenic(LP) variants were identified. Variants of uncertain significance (VUS) were detected, including:

*DSP (c.1235A* *>* *G; p.Q412R)*:Associated with arrhythmogenic right ventricular cardiomyopathy and dilated cardiomyopathy.

*PDE3A (c.2693G* *>* *A; p.R898H)*: Associated with hypertension and brachydactyly syndrome

*PRKCH (c.530C* *>* *T; p.T177M)*: Implicated in cerebral infarction susceptibility

*TTN (c.34402_34416del; p.T11468_V11472del)*: Associated with dilated and hypertrophic cardiomyopathies

Similarly, the brother's genetic testing revealed no pathogenic(P) or likely pathogenic(LP) mutations but identified variants of uncertain significance (VUS) in the *DSP* gene, consistent with the findings in the patient.

The identical presence of this VUS in the asymptomatic brother suggests they may represent benign familial polymorphisms rather than causative mutations, though a modifier role cannot be excluded. Definitive classification requires further segregation analysis and functional studies.

## 3 Treatment

The patient underwent successful implantation of a dual-chamber implantable cardioverter-defibrillator (ICD) for secondary prevention of malignant arrhythmias. Pharmacological therapy was initiated with bisoprolol at 2.5 mg twice daily, titrated to 5 mg once daily after 48 h based on hemodynamic tolerance and heart rate response. This beta-blocker regimen was selected for its proven efficacy in reducing ventricular arrhythmia burden in patients with repolarization abnormalities. Throughout the subsequent hospital course, no recurrence of ventricular tachycardia was observed.

At discharge, the maintained medication regimen consisted of: 1) Bisoprolol 5 mg once daily (for ventricular arrhythmia suppression), 2) Aspirin 100 mg once daily (for antiplatelet therapy).

The patient was enrolled in a structured follow-up program including: 1) Cardiology clinic evaluations at 2 weeks, 3 months, and 6 months post-discharge; 2) ICD interrogation at 1 month and every 3 months thereafter; 3) 24-hour Holter monitoring at the 3 month follow-up visit.

At the most recent follow-up (6 months post-discharge), the patient remained asymptomatic with no documented arrhythmic events on ICD interrogation. The medication regimen was maintained without changes, and no device therapies had been delivered.

## 4 Discussion

Sudden cardiac death (SCD) represents a catastrophic manifestation of various cardiac pathologies, encompassing both congenital and acquired heart disorders. It is strongly associated with, and often preceded by, diverse forms of ventricular arrhythmias (VAs), which constitute a significant contributor to morbidity and mortality related to cardiac rhythm disturbances. The primary etiologies underlying SCD include long QT syndrome (LQTS), Brugada syndrome, and catecholaminergic polymorphic ventricular tachycardia (CPVT), all of which are attributed to dysfunction in cardiac ion-channel proteins that regulate the cardiac conduction system. Notably, these ion-channel abnormalities are not detectable through conventional histological analysis and are typically not associated with structural cardiac pathology that can be readily identified (13).

This case presents a rare and instructive example of ventricular tachycardia (VT) associated with dynamic J-point and ST-segment elevation in lead II, ultimately progressing to torsades de pointes (TdP).

A detailed analysis of the patient's ECG revealed a distinctive pattern of J-point and ST-segment evolution in lead II. Initially, the J point and ST segment demonstrated horizontal elevation (1–3 mm), followed by a gradual transformation into a saddle-shaped ST-segment elevation. Ultimately, the ST segment exhibited a downsloping configuration. These progressive ECG changes were uncorrected and culminated in the development of TdP. Following successful cardiopulmonary resuscitation and stabilization of the patient, persistent ST-segment instability in lead II was observed, manifesting as horizontal depressions, upward-sloping depressions, and horizontal elevations (approximately 1 mm).

The observed ECG evolution provides valuable insights into the electrophysiological mechanisms underlying malignant ventricular arrhythmias. These findings align with current understanding that subtle repolarization abnormalities, often undetectable in standard evaluations, can serve as precursors to life-threatening arrhythmias. The persistence of ST-segment instability in lead II, even after hemodynamic stabilization, further supports the hypothesis of an underlying channelopathy or repolarization disorder.

The characteristic ECG manifestations observed in this case bear resemblance to Brugada waves, which typically appear in leads V1–V3 (14). Similar to the intermittent and variable nature of Brugada waves—where typical patterns may appear transiently before normalizing—this patient demonstrated a gradual evolution from Brugada type 2 (saddle-shaped) to Brugada type 1 (downsloped) morphology (14), with J-point elevation reaching up to 7 mm. Additionally, the ECG exhibited features consistent with lambda waves, a recently recognized marker of abnormal ventricular depolarization and repolarization. Lambda waves (15), characterized by descending ST-segment elevations in the inferior leads accompanied by corresponding T-wave changes and mirror-image patterns in the left precordial leads, have been identified as independent predictors of sudden death risk (16). The coexistence of these electrocardiographic features—Brugada-like waves and lambda waves—in this patient underscores the complexity of arrhythmogenic substrate identification.

The dynamic J-point elevation observed in this case, while reminiscent of classical early repolarization syndrome (ERS), presented a significant diagnostic challenge due to its transient nature and confinement to lead II. A comprehensive differential diagnosis was essential.

Beyond Brugada syndrome, other entities capable of producing J-point abnormalities were rigorously considered: 1) Early Repolarization Syndrome (ERS): Characterized by J-point elevation ≥0.1 mV in ≥2 contiguous inferior/lateral leads, notching/slurring of the terminal QRS, and horizontal/descending ST-segments (3). Arrhythmic risk is influenced by J-wave amplitude, distribution (inferior leads confer higher risk), and dynamicity (17). 2) Acute Myocardial Ischemia/Coronary Spasm: A critical exclusion, as ischemia can cause transient J-wave formation. This was effectively ruled out by normal coronary angiography and a negative acetylcholine provocation test. 3) Other Causes: These include hypothermia (prominent Osborn waves, bradycardia) (18), hypercalcemia (QT shortening, J-notch), and arrhythmogenic right ventricular cardiomyopathy (epsilon waves, T-wave inversion) (19). These were deemed unlikely given the normal metabolic panel, body temperature, and cardiac MRI.

After excluding structural, ischemic, metabolic, and toxicological causes, the most probable diagnosis is intermittent J-wave syndrome with a focal inferior lead manifestation. This is strongly supported by the characteristic dynamic ST-T evolution preceding Torsades de Pointes and the comprehensive negative workup.

Comprehensive diagnostic evaluation, including echocardiography and coronary angiography, conclusively excluded structural myocardial abnormalities and coronary artery disease as underlying causes. This aligns with current guidelines for sudden cardiac death (SCD) investigation, which emphasize the importance of genetic evaluation in cases without apparent structural etiology (20).

The standard four-gene molecular autopsy, encompassing analysis of KCNQ1, KCNH2, SCN5A (associated with long QT syndrome), and RYR2 (linked to catecholaminergic polymorphic ventricular tachycardia), has been expanded to include calmodulin-encoding genes (CALM1, CALM2, CALM3) due to their association with severe, early-onset long QT syndrome (21).

In this case, genetic testing revealed variants in DSP, PDE3A, PRKCH, and TTN genes, none of which have been definitively established as primary arrhythmia-causing mutations. The presence of DSP gene variants in the patient's 53-year-old brother, who exhibited no cardiac abnormalities on echocardiography or cardiac MRI, further complicates the interpretation of these genetic findings. While these variants may represent incidental findings or variants of uncertain significance, their potential contribution to arrhythmogenesis warrants further investigation through additional case reports and functional studies.

The evolving understanding of early repolarization syndrome (ERS) has transformed its clinical significance from a benign electrocardiographic variant to a potential marker of arrhythmic risk. Historically characterized by prominent J waves and concave ST-segment elevation, ERS was traditionally regarded as a normal variant, particularly prevalent (1%–13%) among healthy, asymptomatic young individuals and athletes. However, seminal research by Pieroni et al. in 2008 demonstrated an association between J-wave elevation in inferior and lateral leads and idiopathic ventricular fibrillation or sudden cardiac death (22), fundamentally altering the clinical perspective on this condition. Subsequent case-control and epidemiological studies have further substantiated the correlation between J-wave patterns and unexplained cardiac arrest (23).

The genetic underpinnings of ERS remain incompletely understood, though evidence suggests a heritable component. Functional loss-of-function variants in the SCN5A gene have been identified in 2%–10% of ERS cases. Notably, pediatric cases have revealed KCND3 gene mutations (encoding Ito channels), including duplications and *de novo* missense mutations. Recent genome-wide association studies (GWAS) have identified a single nucleotide polymorphism at the KCND3 marker locus, supporting a potential polygenic inheritance pattern. Additionally, CACNA1C mutations have been implicated in familial ERS with high sudden cardiac death incidence (24). Despite these advances, reproducible evidence for highly penetrant, rare monogenic causes of ERS remains limited.

In the present case, the fortuitous documentation of the complete arrhythmic episode provides compelling evidence supporting a diagnosis of intermittent early repolarization syndrome. The dynamic ECG evolution observed—from initial J-point elevation to malignant arrhythmia development—offers valuable insights into the electrophysiological mechanisms underlying ERS-related sudden cardiac death. This case highlights the importance of continuous ECG monitoring in identifying high-risk ERS patterns and underscores the need for further research into the genetic and molecular basis of this condition.

The limitation should be acknowledged. The ECG changes were mainly recorded in lead II via telemetry, without simultaneous 12-lead Holter monitoring. While this provided valuable temporal documentation, it precluded full assessment of spatial repolarization patterns across all leads. This may reflect either a focal electrophysiological phenomenon or constraints of acute monitoring.

## 5 Future perspectives

The dynamic nature of J-point elevation observed in this case highlights the need for innovative approaches in risk stratification and monitoring,Future studies should prioritize three key directions: developing AI-based ECG algorithms for real-time tracking of dynamic J-point and ST-segment changes; applying functional genomics to clarify the pathogenicity of ion channel gene variants, especially VUS; and establishing multicenter registries to support evidence-based risk stratification using standardized repolarization data. These efforts are essential to enable proactive management in high-risk patients with dynamic repolarization abnormalities.

## 6 Conclusion

This case underscores that dynamic J-point elevation, even when transient or focal, can precede life-threatening arrhythmias. It highlights the critical importance of continuous ECG monitoring in high-acuity settings to capture these elusive electrical precursors. For management, ICD implantation remains the cornerstone of secondary prevention in survivors of idiopathic ventricular fibrillation. Moving forward, proactive beta-blocker therapy and participation in structured follow-up programs are essential components of long-term care for these high-risk patients.

## 7 Patient perspective

The patient has not fully recovered due to his state of consciousness, and his wife, as his legal representative, is fully informed and participates in the decision-making of treatment, understands the alternative treatment measures, and understands the possible adverse consequences of the treatment measures jointly formulated at present, and actively cooperate.

## Data Availability

The datasets presented in this study can be found in online repositories. The names of the repository/repositories and accession number(s) can be found in the article/Supplementary Material.
